# Mental health training for secondary school teachers in Haiti: a mixed methods, prospective, formative research study of feasibility, acceptability, and effectiveness in knowledge acquisition

**DOI:** 10.1017/gmh.2016.29

**Published:** 2017-03-06

**Authors:** E. Eustache, M. E. Gerbasi, M. C. Smith Fawzi, J. R. Fils-Aimé, J. Severe, G. J. Raviola, R. Legha, S. Darghouth, D. J. Grelotti, T. Thérosmé, E. L. Pierre, E. Affricot, Y. Alcindor, M. B. Stack, A. E. Becker

**Affiliations:** 1 Zanmi Lasante, Mental Health and Psychosocial Services, Mirebalais, Haiti; 2 Department of Global Health and Social Medicine, Harvard Medical School, Boston, MA, USA; 3 Department of Psychiatry, Baystate Medical Center, Tufts University School of Medicine, Springfield, MA, USA; 4 Partners In Health, Boston, MA, USA; 5 Department of Psychiatry, Boston Children's Hospital, Boston, MA, USA; 6 Department of Psychiatry, University of California Los Angeles, Los Angeles, CA, USA; 7 Department of Psychiatry, Massachusetts General Hospital, Boston, MA, USA; 8 Department of Psychiatry, University of California San Diego, La Jolla, CA, USA

**Keywords:** Haiti, school-based, task sharing, teachers, training

## Abstract

**Background:**

Engagement and training of educators in student mental health holds promise for promoting access to care as a task sharing strategy but has not been well-studied in low-income regions.

**Methods:**

We used a prospective and convergent mixed methods design to evaluate a customized school mental health 2½ day training for teachers in rural Haiti (*n* = 22) as the initial component of formative research developing a school-based intervention to promote student mental health. Training prepared teachers to respond to student mental health needs by providing psychoeducational and practical support to facilitate access to care. We examined level of participation and evaluated feasibility, acceptability, and perceived effectiveness by calculating mean scores on self-report Likert-style items eliciting participant experience. We examined effectiveness of the training on improving mental health knowledge and attitudes by comparing mean scores on an assessment administered pre- and post-training. Finally, we examined self-report written open-ended responses and focus group discussion (FGD) interview data bearing on perceived feasibility, acceptability, and effectiveness to contextualize participant ratings of training and to identify recommendations for enhancing the utility of mental health training locally for educators.

**Results:**

Mean scores of knowledge and attitudes significantly improved between the pre-test and post-tests; e.g., knowledge improved from 58% correct at baseline to 68% correct on the second post-test (*p* = 0.039). Mean ratings of the training were favorable across all categories and FGD data demonstrated widespread participant endorsement of training acceptability and effectiveness; participants recommended extending the duration and number of training sessions.

**Conclusions:**

Findings support feasibility, acceptability, and a limited scope of effectiveness of brief mental health training for secondary school teachers in Haiti. Further development of approaches to engage teachers in promoting school mental health through training is warranted.

## Introduction

Mental disorders are among the largest contributors to the global burden of disability (Becker & Kleinman, 2013). Mental disorders are also prevalent in youth, and yet the majority of youth with mental illness in low resource settings do not receive care for this condition (Kieling *et al.* 2011; Patton *et al.* 2012). Timely intervention for youth with mental disorders has potentially high return in the prevention of adverse health and social sequelae associated with untreated mental illness (Kieling *et al.* 2011). Expanding access to community-based mental health care and to mental health services for children and adolescents has thus been identified as among the highest of global mental health priorities (Collins *et al.* 2011). Strategies to promote access include service models that extend surveillance, outreach, and delivery into community settings and leverage scarce specialty resources through intersectoral collaboration and task sharing (Kakuma *et al.* 2011).

Task sharing is the reallocation of certain health care related duties from specialized health professionals to health workers and others who have requisite skills but a lower level of training or expertise (Kakuma *et al.* 2011; Padmanathan & De Silva, 2013). Task sharing has demonstrated effectiveness for mental health care (Patel *et al.* 2011; van Ginneken *et al.* 2013) and its optimization for mental health case finding and care delivery has emerged as one of the field's top research priorities (Baingana *et al.* 2015). However, knowledge gaps persist regarding its feasibility and acceptability (Padmanathan & De Silva, 2013). In addition, relatively few studies have examined an extension of the task sharing paradigm to teachers to reduce the burden of mental illness in youth living in low-resource regions (Kakuma *et al.* 2011).

Notwithstanding a dearth of school-based studies of mental health interventions in low- and middle-income countries (LMICs) (Fazel *et al*. 2014*a*, *b*), there are compelling reasons to develop school platforms for community-based mental health promotion. First, school-based health surveillance and preventive interventions are well established for other health conditions (Mnookin 2016). Second, youth with mental health needs are at high risk for non-detection in regions where there is low mental health literacy and where health system access may be constrained by affordability of transportation and opportunity cost for parents. Next, educators have a unique and strategic opportunity to detect behavioral changes in students that could be the first presenting signs of mental illness. For example, teachers have both a proximal and a potentially invaluable longitudinal perspective on changes in social interactions and academic performance that could be the bellwether of an incipient depression or other treatable mental disorder. Finally, teachers have an education and skill set that can be the foundation for recognizing and responding to youth with a mental illness, behavioral problem, or major social stressor that is adversely impacting their functioning at school or their optimal development (Kieling *et al.* 2011).

Haiti is a low-income country where mental and substance use disorders represent an estimated 23% of years lived with disability (YLDs; Vos *et al.* 2015). A major earthquake centered near Port-au-Prince in 2010 resulted in catastrophic casualties and damage, adding to the chronic social adversities Haitian youth already face and straining its health care system, which had already been inadequate to meet mental health needs. Recognizing these unmet needs *Zanmi Lasante* (ZL), a non-governmental organization (NGO) affiliated with Partners In Health (a US-based NGO), launched a plan to expand availability of quality mental health care in its catchment area in Haiti's Central Plateau (Raviola *et al.* 2012, 2013). In addition to enhancing and developing hospital and clinic-based care through psychologist, physician, and nurse training, this plan also expanded community-based mental health services through the training and deployment of a cadre of Community Health Workers (CHWs). Locally referred to as *accompagnateurs*, these CHWs received training in mental health care, specifically through the development of skills to identify people suffering from neuropsychiatric disorders, including depression, psychotic illness and epilepsy, and to provide basic support in the community.

In 2012, development of a complementary school-based platform for community-based mental health promotion was begun through formative research on an intervention entitled the ‘Teachers as *Accompagnateurs* Pilot Study’ (TAPS). The overarching goal of TAPS was to configure the locally familiar *accompagnateur* role (Farmer *et al.* 2001) for teachers within a task-sharing framework, in which they could provide psychoeducation and enrich the psychosocial support network for students as part of a bridge to care between schools and the clinic. Key components of TAPS included delivery of training to secondary school teachers, school-based screening for mental health needs, and facilitation of teacher–student meetings for psychoeducation and other practical support, including navigation to mental health care (see Eustache *et al.* 2014).

The present study evaluates the teacher-training component of TAPS within a framework of formative research aimed at refining its further development (see Gittelsohn *et al.* 2006; Beidas *et al.* 2011). Training was delivered to teachers in advance of the school-based mental health student screening to prepare them for their role as *accompagnateurs* in meeting with students. In this role, teachers were to respond to student mental health needs with psychoeducational and practical support, including by facilitating access to care when appropriate. Because the future utility of their *accompagnateur* role was predicated, in part, on their knowledge of manifestations and impact of mental illness in youth as well as locally available treatment options, this training was developed to augment teachers’ professional expertise with specific knowledge and skills relevant to school mental health in Haiti. This study aimed to evaluate the feasibility and acceptability of this scope of training content and format of delivery to teachers as well as its effectiveness in improving knowledge and attitudes relevant to school mental health.

## Methods

We used a prospective and convergent mixed methods design to assess feasibility, acceptability, and effectiveness on short-term relevant knowledge gain of a brief mental health training for secondary school teachers in Haiti's Central Plateau. The delivery and evaluation of this mental health training was a component of formative research on a school-based pilot mental health intervention, TAPS, described elsewhere (Eustache *et al.* 2014). This study protocol was approved by the Harvard Medical School Office for Research Subject Protection and the ZL Ethics Committee. Written informed consent was obtained for each study participant. Study findings reported here are based on data collected in 2013.

### Study sample

Teacher study participants were selected purposively by nomination by their respective school principals at each of four participating schools. Schools were selected both for convenience and purposively for location within the ZL catchment area in Haiti's Central Plateau and to comprise both public and independent schools. The participation rate was 91.7% with 22 (18 male, four female, mean age = 40.1, s.d. = 7.16) enrolled of 24 eligible teachers nominated. It should be noted that the majority of teachers in the school system are male.

### Procedures

Study procedures aligned with aims of the broader pilot study intervention by providing teachers with information and principles relevant to school mental health, in order to prepare them for their study-specific role in the ensuing pilot intervention (Eustache *et al.* 2014). This role, as ‘teacher-*accompagnateur*,’ was to provide students with guidance and support that could facilitate their access to local mental health services. Study procedures were developed following consultation with Haitian educators, recruited independently of the teacher participants described in the present study, via focus group discussions (FGDs) about perceived mental health needs and engagement of educators in a school-based intervention. Participants were provided with meals and lodging during the training, but did not receive financial compensation for participation.

#### Content and delivery of the training curriculum

Study participants attended and completed a single training session lasting 2½ days that included didactic presentations, interactive discussions, and role-plays. The teaching was primarily led by study investigators who were also mental health clinicians at ZL and conducted in Haitian Creole and French. This training was delivered just prior to the start of the academic year.

The training curriculum was adapted from existing training resources for community health workers at ZL and augmented with content developed by study investigators to enhance its relevance to school mental health. The curriculum was organized around a framework of ‘the four Rs’ – recognize (*rekonèt*), respond (*reponn*), refer (*refere*), and [build] resilience ([*bati*] *rezilyans*) – intended to provide a foundation of mental health knowledge relevant to teachers’ routine role as educators and also to their study role as teacher-*accompagnateur*. Specific topics included: child and adolescent development; signs and symptoms of major mental disorders in adolescents and adults; an overview of effective treatments for mental disorders; recognizing a psychiatric emergency; approaches to responding to students with mental health needs; local mental health resources available to students for referral; stress management; Haitian mental health law; and promoting resilience through support of adaptive coping strategies. This curriculum was summarized and distributed in a 118-page manual written in Haitian Creole (available upon request); an English language version of the table of contents is displayed in the appendix. Corresponding slide sets were developed, also in Haitian Creole, to facilitate learning and discussion during presentations.

### Assessment of feasibility, acceptability, and effectiveness in promoting knowledge gains

#### Quantitative assessments

As a proxy for feasibility of training, we examined level of teacher participation by calculating rates of study participation, completion of training, and response to assessments. In addition, we developed two kinds of written quantitative assessments to examine training feasibility and acceptability, as well as its effectiveness in improving relevant knowledge and attitudes. These assessments were translated from English into Haitian Creole, back-translated, and adjusted for syntax and accuracy after comparison with the original version.

##### Mental health knowledge and attitude assessment

We used an unstandardized written assessment as our primary measure of mental health knowledge and attitudes at baseline and at two post-training time points. We developed the assessment for this study in order to align item content with training curriculum content and optimize its relevance to the local social context. Participants responded to the same assessment of mental health knowledge and attitudes at baseline (Time 0), immediately post-training (Time 1), and approximately 6–9 weeks following training (Time 2). Fund of relevant mental health knowledge assessment comprised 48 multiple choice items. Attitudes toward mental illness and its treatment comprised 40 additional Likert-style items on a five-point scale, ranging from ‘Strongly disagree’ to ‘Strongly agree.’ Internal consistency reliability for this latter measure was acceptable (Kline, 1999) across administrations: Cronbach's *α* at Time 0 = 0.69; Time 1 = 0.84; Time 2 = 0.81.

##### Participant feedback about the content, delivery, and utility of training

**Fig. 1.**
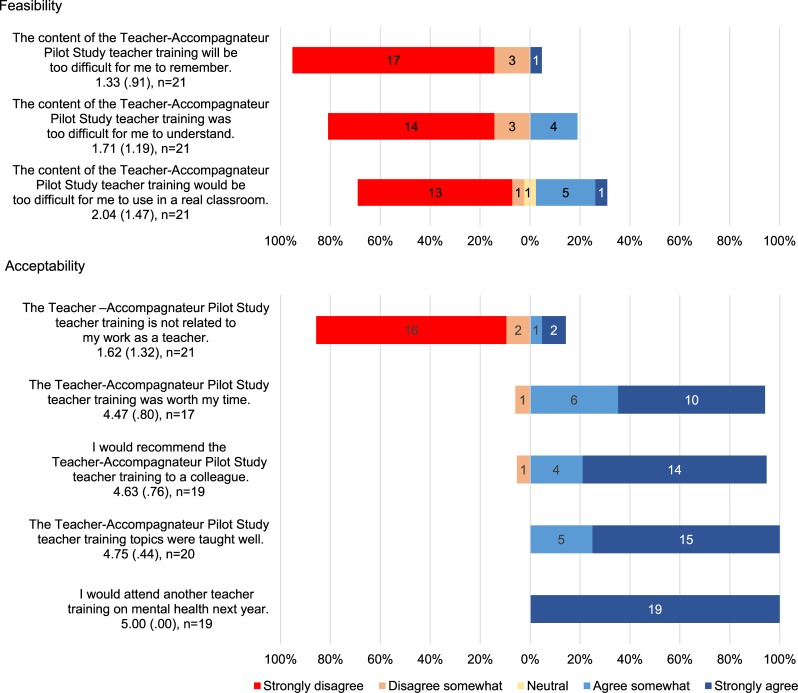
Post-training (Time 1) feasibility and acceptability. Response options were Likert-style and ranged from 1 (Strongly disagree) to 5 (Strongly agree).

We assessed perceived feasibility and acceptability of the training immediately after the first post-test with eight Likert-style items, on a five-point scale, ranging from ‘Strongly disagree’ to ‘Strongly agree.’ Feasibility was operationalized as perceived level of difficulty of the training and acceptability was operationalized as user satisfaction (three and five questions, respectively, displayed in Fig. 1; see Padmanathan & De Silva, 2013).

#### Qualitative assessments

##### Written self-report open responses

Immediately following the training, participants responded to nine open-ended questions regarding their experience of training, with specific items asking about user satisfaction with the training delivery and relevance of content, level of difficulty, and recommendations for improvement (Table 1). Of these, two questions elicited feedback specific to duration and periodicity of training. Responses were translated into English for coding and analysis.

**Table 1.** Teacher perceptions of feasibility, acceptability, and effectiveness conveyed in post-training written open-ended responses

Selected probe questions^a^	Thematic domains	*n* (of 22) endorsing theme	Selected responses illustrating key themes
Acceptability			
What training activity did you like the most? Why?	All activities	12	‘I liked the role plays the most, because they showed us the reality we are going to face in the classroom.’ ‘All the activities were really important, and they will help us to address our responsibilities as teachers.’ ‘I cannot say what I enjoyed the most I had the same enthusiasm I started the training with, is what I ended it with.’ ‘I like the evaluations (pre- and post-tests) because they allowed me to measure what I learned. I memorized them and I will be able to help those who have issues.’ ‘I liked all the activities of the training. They enabled me to understand the behaviors of the people in my immediate circles. It also helped me more quickly understand the students that have mental health issues, the way to approach them. This training helped me understand human reactions better.’ ‘I liked all the activities pertaining to the training because they will help me care better for my classroom.’ ‘I liked all the activities because they helped me develop my skills and knowledge further.’ ‘I liked all the activities of the training. I had fun.’
	Training addressed teachers’ responsibilities and experiences	8	
	Role-playing or other specific activity	8	
	Presentation quality or style	3	
	Training helps outside of school	2	
	Accommodations	1	
	No response	1	
What training activity did you like the least? Why?	Resulted in fatigue or tiredness	7	‘There are no activities that I disliked. They were all important to me.’ ‘We spent too much time on certain topics, which meant that after a while, we could no longer focus.’ ‘What I disliked is that sometimes we spent too much time seated. From a pedagogical perspective, it is recommended to take a break every 3 h. The memory otherwise gets tired.’ ‘We had too many themes during the 2½ days. Especially during the second day, during which there was no time left for questions.’ ‘The activity I enjoyed the least came from my colleagues, when they went for food those who were ahead took too much and those who were behind got nothing.’
	No least favorite/liked them all	6	
	No response	6	
	Manners comment	3	
Acceptability and effectiveness			
What did you learn that was valuable and that you will use in your work?	4Rs or how to approach/ help students	13	‘I learned many things, like signs and symptoms of mental illnesses, ways to approach mental illness in the classroom reality, and other theories about psychology.’
	‘All’ or ‘many’/ list of several	12	‘All the goals we worked on during the training were really important. I will use all of my effort to use them as a teacher in my school, in favor of the kids.’
	Better understanding of mental health issues, symptoms	6	‘I learned the way in which teachers can approach problems students can encounter and the appropriate ways to handle them.’
	Classroom management	4	‘Everything I learned is important to me because I didn't have much information about it before. I would like to use all the new knowledge in my work.’
	Training helps outside of school	4	‘I must admit that I was not used to helping students with mental health symptoms but now, I will do it regularly in the event of a crisis, or epilepsy or depression.’
			‘What has value for me is the way in which I must approach the students who have issues.’ ‘Everything you presented had value for me and I will use it to help my family and all the other people I will be around to help.’ ‘I know now when a child exhibits one or more symptoms of a mental health disorder. I know which behavior to adopt, how to approach him, how to engage the conversation to promote his trust. I learned the four Rs.’ ‘Everything I learned is very important to me because in the community where I live, the knowledge will be useful to me and to others too. Especially the four Rs.’ ‘Everything I learned has a lot of value. I will now find out how to apply it in classroom activities.’ ‘I learned the four Rs which are recognize, respond, refer and resiliance that I will use in my work to help students with mental health disorders.’ ‘I learned how to manage students with stress and mental health problems.’ ‘I learned many things, for instance, the numerous signs and symptoms in a depressed person […].’
What information was included that you do think will NOT be very relevant for teachers?	All information useful/none was irrelevant	17	‘From my point of view, all the information was useful to the teachers because even though we had a small piece of knowledge about mental health it was not enough for us to do what we have to do normally. It's true we cannot say that we are very good, but there is another extra drop of water that has fallen into our reservoir of knowledge.’ ‘All the information is useful to teachers, as it pertains to information they encounter on their path.’ ‘All the information will be useful to the teachers because it is a tool to understand the students’ issues.’
	No response	4	
	Manners comment	1	
Was there anything you did not understand? Please give specific examples.	Everything understood/ nothing not understood	12	‘I understood everything because the facilitators did the work well and they used clear language.’ ‘I understood everything and especially the role plays.’ ‘There is nothing that I did not understand. But it would be even better that the facilitators think about refresher courses/sessions in a while.’ ‘Not really. Everything was clear to me. You know, when you receive training in a field that is not yours, clarity comes progressively.’ ‘I did not very much understand when you talked about defense mechanisms, such as denial, and so on.’ ‘I did not really understand how we can detect depression.’ ‘What was not clear to me was the word depression, often employed by people when they say ‘I have depression.’ I asked the question, but I did not get an answer.’ ‘The definition of certain acronyms, such as PTSD.’
	Information/clarification on mental illnesses	6	
	No response	3	
	Communication issues or questions	2	
	Intervention methods or logistics	2	
	Suggested refresher	1	
Feasibility and acceptability			
What are your recommendations to improve this training? What would you change? (For example, what activities, illustrations, etc. would you change?) What additional comments do you have?^b^	Continue as is, continue but add refreshers, lengthen and add content, or expand	16	‘Extend the pilot study to all the schools in the country.’ ‘I ask that you continue this initiative.’ ‘Please continue with this kind of training, and I wish you courage. Thank you.’ ‘I thank you for taking this initiative and I would like that you continue this work, and that you conduct more research in this field in order to be even more effective.’ ‘I recommend that we expand this training to new levels of schooling, like pre-school and grade school, secondary, and even for the teachers who already got some training today.’ ‘I would like that this training be extended to all the country's domains of work, such as the police. So when someone with mental health issues but no one to support him/her can find support/help everywhere else.’ ‘Even though this is a pilot study, I wish there had been more teachers participating, and from more schools.’ ‘Considering all of the problems in the education system in Haiti, mostly all of the problems related to mental health for students, I'm asking that the program could reach more schools, more teachers could be trained in that field.’ ‘I wish courage to all the facilitators, and I wish the leaders of the program could show a lot of interest for the other schools that have not been reached by this program.’ ‘My recommendation would be to have ongoing training to make the work already done more efficient.’ ‘I believe that this training is not only good, but also important and efficient. I would like for you to research how to continue supporting this country's teachers, faced with such natural disasters. Thank you and many congratulations to Ekip Wozo.’ ‘Congratulations to all the trainers. My wish would be to devote more time to training and for the training to continue and extend to all schools in the country. Parents should also receive information, possibly use radio or TV broadcasts.’
	Thank you/congratulations offered to team	9	
	Incentive or support for teachers	7	
	Lengthen, add more time for existing content	5	
	Circulate the day's agenda	3	
	More interactive	2	
	Accommodations	1	
	Add more content, no mention of adding time	1	
	Increase variety of activities	1	
	Future participation	1	
	Avoid fatigue or tiredness	1	
	Include psychiatrists	1	
	Manners comment	1	
			‘I am satisfied with the training, and I will add that I would like to become a social worker or psychologist to be able to help you. I thank Harvard University and Zanmi Lasante. May God continue to help you. However, just one more thing I'd like to say is that I think teachers could have been given [financial] compensation.[…]’ ‘I think for such important work, the teachers lack the means to achieve the goals. We should have more means to accomplish the goals and more motivation.’ ‘This training demands a lot more days. Instead of two and a half days, at least a week.’ ‘Give the daily agenda, provide beer for those who can drink, and per diem.’ ‘I think that everyone understands the importance of time for teachers. We will need to allow the teachers to find more time to continue the good work.’ ‘I would like to add that the program would succeed if it expanded. Also, in the case of travel, it would be good if the facilitators cover some of the costs to the teachers since they are away from their family.’

a Data presented in table exclude two questions specific to duration and periodicity.

b Responses from these two questions were combined for tally and illustrative excerpts.

##### FGD interviews

After completion of the subsequent school-based intervention phase (corresponding to Time 2, at 6–9 weeks following training) – we conducted two FGD interviews with a convenience sub-sample of teacher study participants (*n* = 12) to debrief them on their experience in participating in the study and elicit their ideas for optimizing this school-based intervention promoting student mental health. For example, interviews included the following prompts: ‘Please share your perspective on how well this program met objectives to train teachers to support student mental health?’; ‘Please share your perspective on the challenges teachers faced in participating in TAPS’; and ‘Based on your experience, what ideas do you have to make this program more effective for teachers in the future?’ FGDs were facilitated by two study investigators in Haitian Creole and were audio-recorded. Interviews were transcribed and translated into English for analysis. A second bilingual investigator verified the accuracy of the transcription and translation.

### Data management and analyses

Self-report data were entered into an Excel file and verified. For written open-response items, missing responses were coded and tallied as ‘no response.’ For other written self-report data, missing and double entered responses were identified and addressed as follows. Knowledge assessment responses were scored dichotomously as correct or incorrect: missing and double-entered responses were coded as incorrect. Participants missing more than 10% of attitude test responses at any one of the time points were excluded from analysis of that measure. For participants missing fewer than 10% of attitude responses, the mean of all answered items was calculated. This procedure resulted in an analysis sample of *n* = 22 for knowledge assessment and *n* = 15 for assessment of attitudes. Missing data for all other measures were addressed item-wise, with sample sizes shown in Fig. 1.

#### Statistical analysis

We assessed mental health knowledge at baseline (Time 0), immediately post-training (Time 1), and 6–9 weeks post-training (Time 2) by calculating a score based on the percent correctly answered at each assessment. Likewise, we assessed mental health attitudes at Time 0, Time 1, and Time 2 by calculating mean scores of that component of the assessment at each time point. We evaluated effectiveness of the training in improving mental health related knowledge and attitudes with repeated measures analysis of variance (ANOVA) and follow-up paired sample *t* tests. Prior to performing these analyses, we tested for normality in mental health knowledge and attitudes at each time point, with Shapiro–Wilk tests. As the distributions of mental health knowledge and attitudes did not significantly differ from normal (all *p* values >0.05), parametric tests were used.

We assessed teachers’ perceived feasibility and acceptability of the training by calculating mean scores for each Likert-style question from the participant feedback evaluation at Time 1. Quantitative analyses were conducted in SPSS 23 (IBM Corp, 2015).

#### Qualitative data analysis

We evaluated participants’ written open-ended responses by first generating a list of themes in response to each question; these themes were either structured *a priori* in alignment with study aims or identified inductively from the responses so as to inform an understanding of teacher experience and recommendations relevant to mental health training. Two independent raters then assigned codes linking responses to these thematic categories. These coding assignments were compared to identify discrepancies, which were then resolved by discussion and refinement of thematic categories with a third coder. A final set of themes was generated; this set comprised both themes specific to particular questions, as well as themes that related to one or more questions. Coding was finalized by consensus among the coders. We then generated summary data regarding the frequency of theme endorsement in each category and selected illustrative responses for presentation. However, responses to the two items regarding duration and periodicity of training were tallied separately and later combined with relevant responses across categories in descriptive summary data.

Whereas the scope of the FGDs was broad and covered the entire TAPS pilot intervention in addition to training, for the present study, two study investigators reviewed the FGD transcripts independently to identify broad themes relating to the goals of evaluating the training. The readers then independently coded responses corresponding to these themes, next compared coding for discrepancies, and subsequently reconciled these discrepancies by consensus and within the context of the overall transcripts. We tallied the number of respondents making comments within each of the coding categories and selected excerpts that further contextualize quantitative data for presentation.

## Results

### Feasibility and acceptability

Feasibility of recruitment of teacher participants in a single-session mental health training is supported by high rates of participation, completion of training, and response to the assessment. The participation rate was 91.7% among eligible teachers nominated. Moreover, 100% of enrolled participants completed the training and responded to pre- and post-test assessment measures at all three timepoints.

In addition, mean scores of participant responses to Likert-style questions about the level of difficulty of the material support that respondents perceived the training as feasible to learn and apply in a classroom setting (Fig. 1). Responses to additional Likert-style questions regarding satisfaction with training likewise support that participants perceived the training as acceptable. Mean scores reflect somewhat to strong agreement with statements that the training was well taught, worth their time, and that they would recommend it to colleagues; all responding participants indicated strong agreement with their willingness to return for future training. Figure 1 displays specific values and their distribution.

### Effectiveness of training in improving mental health knowledge and attitudes

**Fig. 2.**
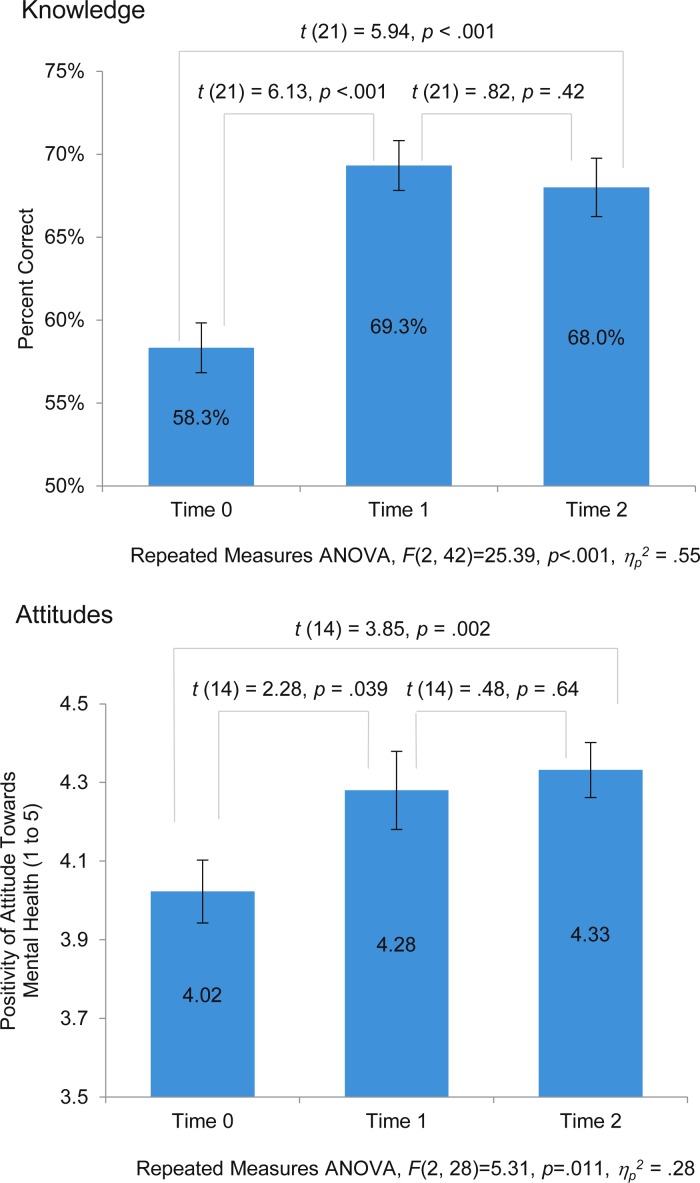
A comparison of mental health knowledge (*n* = 22) and attitudes (*n* = 15) at baseline (Time 0), immediately post-training (Time 1), and 6–9 weeks post-training (Time 2).

Repeated measures ANOVA showed a significant change in both measures of knowledge and attitudes across the three test administrations (*p* < 0.001 and *p* = 0.011, respectively). Figure 2 displays additional relevant statistical values. Follow-up paired simple effects *t*-tests of pre- and post-test scores indicate that teacher knowledge and attitudes about mental disorders improved significantly after training, with effect sizes that can be interpreted as large or medium in magnitude, respectively (*p* < 0.001, Cohen's *d* = 1.32; and *p* = 0.039, Cohen's *d* = 0.60; Cohen, 1988). Knowledge increased by 18.8% of the pre-test score, positive attitudes toward mental health increased by 6.4% from the baseline attitude score, and these improvements were retained through the conclusion of the ensuing school-based TAPS intervention (Eustache *et al.* 2014). Time 2 scores were significantly higher than pre-test scores, with large effect sizes for both (*p* < 0.001, Cohen's *d* = 1.28; and *p* = 0.002, Cohen's *d* = 1.00 for knowledge and attitudes respectively; Cohen, 1988). Finally, there was no significant decline in knowledge (*p* = 0.42, Cohen's *d* = 0.14) or attitudes (*p* = 0.64, Cohen's *d* = 0.12) between measures recorded at post-tests at Time 1 and Time 2.

### Perceived feasibility, acceptability, and effectiveness in post-training feedback

Teacher participant responses to narrative-style open-ended prompts strongly support that teachers perceived this training to be feasible, acceptable, and effective in improving their knowledge of mental health (see Table 1). Of particular importance to the goals of the training, each of the teachers noted its value and relevance for their professional responsibilities, including their work with individual students and in classroom management. Although not the purpose of the training, nearly one-quarter commented that the training would be also valuable outside of their professional responsibilities (*n* = 5, 22.7%). The strong endorsement of the perceived value of the program is perhaps most clearly evidenced in the nearly unanimous (20 of 22 participants; 90.9%) recommendation, across the open-ended responses, that mental health training be continued and/or expanded in some way.

Notwithstanding the enthusiasm for the training and affirmative feedback, teacher responses also indicate important areas for improving teacher experience and the potential impact of the training. For example, nine respondents (40.9%) indicated tiredness or fatigue across at least one of their open-ended responses. Approximately half of teacher participants (*n* = 10, 47.6%) indicated a preference for periodic training, whereas approximately one-quarter of participants (*n* = 6, 28.6%) preferred a single session format. The majority of teacher participants indicated support for increasing the duration of the teacher training, as 14 (63.6%) indicated they would prefer a longer training as compared with only five (22.7%) indicating that the current length was appropriate. Notably, none of the respondents indicated a preference for a shorter training. Importantly, approximately one-third of the respondents (*n* = 7) recommended financial or other support for teacher participation in the future. Finally, four participants (18.2%) noted inappropriate manners from the facilitators or fellow participants (see Table 1), indicating opportunities to improve the experience by greater attunement to local sensitivities.

### FGD debrief of participant experience relevant to the mental health training

Focus group discussion data collected after the implementation phase of the study also strongly support acceptability and perceived effectiveness of the training in promoting a better understanding of mental health among teacher participants following the opportunity to apply it in the school setting during the study. We identified and coded transcripts for statements illustrating three relevant themes: (1) affirmation of acceptability (e.g. comments that training was important, needed, or relevant); (2) affirmation of effectiveness (e.g. tangible benefits perceived to be linked to training); and (3) recommendations for improving the feasibility and/or utility of future iterations of training. We did not identify any statements recommending discontinuation or diminution of the scope of training.

It is noteworthy that every FGD respondent (*n* = 12 of 12) made at least one statement endorsing the perceived value of the training and 9 of 12 made a comment endorsing its acceptability. Perceived benefits included appreciation for skills in improved classroom management, building rapport with students, and conceiving of a non-punitive approach to certain unacceptable behaviors that they could now understand as related to emotional disturbance. Each of the FGD participants also made recommendations relevant to improving the feasibility and/or utility of training. Suggestions included providing concomitant training for school principals and extending the content taught and extending the training to other school teachers in Haiti. They also recommended that recognition and/or financial support for future teacher participation in training would be valuable. Selected excerpts from comments illustrating both positive experience with and regard for the training as well as recommendations for improving future iterations are displayed in Table 2.

**Table 2.** Teacher participant experience and recommendations regarding acceptability, feasibility, and effectiveness of training

Theme *n* (of 12) endorsing	Selected excerpts to illustrate key themes^a^
Acceptability (*n* = 9)	[ … ] So I feel that the training is important and that it is relevant and it is a need, it is so much of a need, this training is so important to me, I feel unsatisfied, I feel unsatisfied because there are so many people who are waiting for it, the next step should be already in place. [short interjection by each facilitator] In general then, I can say that I feel proud, I feel very happy, the training helped me grow and I feel I am transformed every day that goes by. [ … ] [ … ] We feel that it was the right time to have this training for all the teachers, because through our research and all we came to understand from this training, we found it successful. [ … ] [ … ] this training was premier and important too, I have to tell you that I am a rigid teacher and when I was teaching, I used to act in a certain way, but I have to tell you from this training I discovered a lot of things that I can do while I'm working with the students, this training helped me in a way that when the students will have a reaction, I quickly refer to the training I got and look for the motive of his or her behavior, it was a good experience for me.
Effectiveness (*n* = 12)	[ … ] and I must tell you that it is the first time, in the Department Centre [Central Haiti], that a training on mental health was organized for teachers, and what we have noted, is that people can have all sorts of problems, like stomach problems, foot problems, but mental health is never taken into account, and Zanmi Lasante through Harvard University, provided this training for us, and we ourselves, we were able to be reached, at the school level, I can say, that plays an essential role, due to the fact that we ourselves, could not easily in the past identify people who are suffering from mental issues, but now, with this training that we received, it enables us to work productively on mental health issues [ … ] As for me, the training really helped me because it enabled me to change my behavior with the children, and even in my own environment I try to understand other people, all those I interact with daily, and also this training is targeted, it targets the teachers that are in contact daily with the students and you are managing crowds, it helps me manage the class differently and also helps me understand when children react in a certain way in the classroom, it is because they are facing a problem that makes them react the way they do, and it helps me approach the child and ask questions [ … ] [ … ] But today, after I have received the training with the WOZO team –Zanmi Lasante and Harvard, I have become a more moderate teacher, why? Because I know that the student sometimes presents or displays behavior because something [not good] out of the ordinary happened, either at home or maybe within his environment and he has no way he can fix it and he has no other way to make others aware of it except through this action. [ … ] I want to say this training was very useful, we learned a lot, it transformed us from the way we used to manage the students. We used to have a way of managing problem cases; since the training we deal with them another way. I have to tell you that there are some children that were misbehaving, we had to expel them but after this training we saw it was not the best solution, all this is to show you how this training was useful. [ … ]
Recommendations (*n* = 12)	[ … ] Yes the training was really good for us as teachers and as parents, and I would like Harvard University and Zanmi Lasante to go further with us with the training. According to me what I would like to be achieved is to make the program more efficient for teachers, that is to increase sessions of training and then teachers will have more skills. They will know more about how to address a case when it happens, so now we are voting for continued training. [ … ] Now in regard to what we wish for the success of this training, we notice that this as both a study and training that we got was a little intense, honestly there are some concepts that we did not learn well enough. What I would suggest is to try to do this training over time so that we could really master the concepts, thanks. [ … ] So we encourage this work and ask, as my other colleagues already said, that the work continues, and that it could be expanded through the whole country. [ … ] I would like for Harvard University jointly with Zanmi Lasante to do training for the [supervisors,] disciplinarians, [and] principals [ … ]

a Some excerpts encompass text beyond the coded theme to provide context; some excerpts encompass more than one theme.

## Discussion

Study findings support the feasibility and acceptability of a brief, introductory single session, multi-day training on mental health for secondary school teachers in Haiti at small scale. Findings also support that training resulted in improved mental health knowledge and attitudes over the duration of the study. Specifically, high rates of participation, training completion, and response to pre- and post-test assessments support feasibility of recruiting and engaging secondary school teachers in Haiti in mental health training. Moreover, interval changes in pre- and post-training assessments demonstrate that teachers’ mental health knowledge and attitudes significantly improved after training and that these gains were retained at Time 2, following the school-based intervention when participants had an opportunity to act as teacher-*accompagnateurs* (Fig. 2). The effect sizes relating to the improvements from baseline (Time 0) to the final post-test (at Time 2) were large (Cohen, 1988). The improved performance is consistent with our expectation that the content focused on recognizing, responding to, and referring students with mental health needs as well as building resilience for mental health would comprise an appropriate scope for education professionals, while also introducing new information relevant to school mental health.

Additionally, teacher participant feedback about the training was highly positive. Quantitative proxy measures of feasibility and acceptability yielded mean scores uniformly in the favorable range. These positive ratings were complemented and corroborated by qualitative data both at the completion of training and also after an opportunity to implement it in the school-based pilot intervention (TAPS). That the vast majority of respondents recommended continuation and/or expansion of the training, and also that none recommended abridging or discontinuing the training, reflects their positive regard for its value. Next, these FGD findings provide a contextualized understanding of how teacher participants valued their training and its relevance to their work.

Successful delivery of mental health training to teachers in Haiti not only provides a potentially novel and important resource for their professional development, but also can set the stage for their promotion of youth mental health in several key ways. Beyond the rather modest aim of the training to support teachers in helping students navigate mental health services, our pilot data suggest other fruitful positive impacts of engaging teachers in mental health awareness and promotion. Our respondents gave examples of how they perceived this brief mental health training had a positive impact on their capacity to support individual students, manage the classroom, and identify school disciplinary practices that could be improved to better respond to student needs. Although other task-sharing models for mental health delivery, including those involving teachers, have largely focused on equipping non-health professionals to implement a specific psychosocial intervention or teach a mental-health based or enriched health curriculum, this study describes an additional avenue for partnering with teachers in promoting youth mental health, which is based on the locally established model of *accompagnateur*.

The unmet treatment needs associated with mental illness impose vast health, economic, social, and human costs globally (Becker & Kleinman, 2013). Closing the treatment gap by expanding access to quality mental health services is a cardinal challenge for health systems in LMICs (Baingana *et al.* 2015). Although task sharing with community health workers is among leveraging strategies to meet workforce shortfalls in mental health care (Becker & Kleinman, 2013), there are comparatively few studies addressing task sharing with teachers. In addition, few data are available to illuminate how the prospective health task-sharing workforce perceives the feasibility and acceptability of this role (Padmanathan & De Silva, 2013). Among numerous challenges identified, lack of competencies and training and increasing workload (Padmanathan & De Silva, 2013) are especially salient considerations for the utility and sustainability of mental health task sharing with teachers. Even though another post-earthquake initiative to provide Haitian teachers with mental health training was reportedly well-received (St. Louis, 2014), school-based health interventions integrating teacher contributions arguably add to an already considerable teacher work burden (Rajaraman *et al.* 2012; Fazel *et al.* 2014*b*).

Whereas study findings support the feasibility, acceptability, and potential benefits of this approach to delivering training on mental health to a highly select group of secondary school teachers in Central Haiti, they also point to opportunities to optimize this training approach further. Notably, participant recommendations for additional logistical support (to underwrite their time for training), engagement of school leadership in supporting teachers’ application of skills in the classroom, and extension of the training beyond a single session are consistent with empirical evidence for practices that can improve effectiveness of training (Fixsen *et al.* 2005). Training programs have been shown to significantly increase teacher knowledge over baseline in previous school-based mental health interventions (Owens *et al.* 2005; Jorm *et al.* 2010). Training is recognized as an efficient means of introducing knowledge, background, and skills requisite to implementation; however, transfer and successful implementation of new skills is optimally achieved by both training *and* coaching (Fixsen *et al.* 2005). In FGD interviews, teachers indicated that the opportunity to deploy some of the skills in the context of the ensuing study was useful. Although guidance was offered on demand during the subsequent TAPS intervention, the incorporation of supervision into future iterations of this training would likely enhance the competencies in the application of this knowledge to classroom settings. In addition, although school administrators’ support for the project was sought at the onset of TAPS, their additional engagement throughout teachers’ deployment of skills learned in training would likely promote the implementation of these skills (see Fixsen *et al.* 2005).

Strengths of the study include its prospective and convergent mixed methods design, including quantitative assessment of changes in knowledge and attitudes regarding mental health and qualitative assessment of perceived benefits and weaknesses of the procedures and impacts of training. There were also several limitations of this pilot study. The study sample was small and purposively selected for teachers nominated by their respective principals; thus study participants may have had greater interest in school mental health than their counterparts and generalizability of findings to other teachers and settings may be limited. That being said, study findings also point to the future potential value of extending the reach of this type of training to a broader base and on a larger scale. Because we tailored knowledge and attitude assessments to the specific needs of this formative research, our assessment of improved knowledge and attitudes was conducted with an unstandardized measure and has unknown construct and predictive validity. Use of standardized measures in future development of evaluation of this training will be useful in benchmarking knowledge improvement against other training approaches (Beidas & Kendall, 2010). In addition, a large number of missing responses on our attitudes measure resulted in exclusion of seven participants from the corresponding analyses of change over time. Future evaluations should seek to elicit responses from all participants, especially since this may have introduced bias. Moreover, social desirability may play a role in our qualitative and quantitative results. However, it is worth noting that teachers reported both constructive criticism as well as positive feedback, suggesting that social desirability would not be the only factor driving their responses. Evaluation of effectiveness of the training was limited to short-term acquisition of mental health knowledge and attitudes. Although we reported teachers’ perceptions of how they applied the knowledge acquired from training, we did not evaluate behavioral measures of participants’ application of knowledge and skills covered during the training, and our findings do not illuminate the impact of this training on actual classroom practice. Indeed, acquisition of knowledge is necessary, but not sufficient, to ensure teachers would demonstrate competency as teacher-*accompagnateurs* (see Fixsen *et al.* 2005; Lyon *et al.* 2011). Future development of mental health training should therefore incorporate supervision and coaching as trainees practice applying new knowledge and skills in this school-based setting as a formalized element of the training process.

## Conclusion

The health burdens of mental illness in LMICs among children and adolescents are high and youth are especially vulnerable to social structural barriers to care. A timely investment in mental health in adolescents and transitional age youth may mitigate adverse social consequences of mental illness as youth transition from school into the workforce or higher education. Yet, engagement of educators through task sharing that can facilitate youth access to clinical services has not been adequately explored in the scientific literature. We found support for the feasibility and acceptability of a pilot brief mental health-training program for teachers in Haitian secondary schools. We also found that training was effective in improving short-term gains in relevant knowledge and attitudes, a prerequisite for its application in practice. Notably, teacher participants perceived associated benefits outside of the initial scope of the program that extended to potentially positive impacts on classroom management and interest in promoting school policies that support student well-being. Notwithstanding the limited generalizability from a select group of possibly highly motivated teachers, both quantitative and qualitative study data support that mental health training would likely be well received if developed further and extended to other secondary school teachers in Haiti. We found that teachers were enthusiastic, active, and successful participants with respect to understanding mental health and its relevance to their students and to their role as educators. We underscore our finding that teacher participation in future training could be better supported by financial resources to underwrite the associated opportunity and other costs incurred by attending a training (e.g. a per diem for participation would be appropriate in Haiti) as well as by broader engagement of school leadership.

## Appendix

**Introduction to Mental Health: A Resource for Teachers**

**Table of Contents**
